# Transarterial chemoembolization as an alternative to radioembolization is associated with earlier tumor recurrence than in radioembolization-eligible patients

**DOI:** 10.3389/fonc.2023.1081479

**Published:** 2023-02-28

**Authors:** Sung Won Chung, Heejin Cho, Hyunjae Shin, Jeayeon Park, Ju Yeon Kim, Ji Hoon Hong, Moon Haeng Hur, Min Kyung Park, Yun Bin Lee, Su Jong Yu, Myungsu Lee, Yoon Jun Kim, Jin Chul Paeng, Jung-Hwan Yoon, Jin Wook Chung, Jeong-Hoon Lee, Hyo-Cheol Kim

**Affiliations:** ^1^ Department of Internal Medicine and Liver Research Institute, Seoul National University College of Medicine, Seoul, Republic of Korea; ^2^ Department of Radiology, Seoul National University College of Medicine, Seoul, Republic of Korea; ^3^ Department of Nuclear Medicine, Seoul National University College of Medicine, Seoul, Republic of Korea

**Keywords:** transarterial radioembolization (TARE), transarterial chemoembolization (TACE), hepatocellular carcinoma, lung shunt fraction (LSF), time to progression

## Abstract

**Introduction:**

Although transarterial radioembolization (TARE) using yttrium-90 (^90^Y) is a treatment option for large hepatocellular carcinoma (HCC), a fraction of patients are ineligible for TARE due to high lung shunt fraction (LSF).

**Methods:**

We evaluated if treatment with transarterial chemoembolization (TACE), owing to TARE ineligibility was associated with early HCC progression. Consecutive patients with HCC who were initially TARE candidates were included. Patients with vascular invasion or metastasis were excluded. Primary endpoints were time-to-progression (TTP) and overall survival (OS). The secondary endpoint was objective response rate.

**Results:**

In total, 175 patients were included: 144 underwent TARE (TARE-eligible group) and 31 underwent TACE due to high LSF (TARE-ineligible group). This latter group had larger tumors (13.8 cm *vs*. 7.8 cm, *P*<0.001) and higher MoRAL scores (1,385.8 *vs*. 413.3, *P*=0.002) than the TARE-eligible group. After balancing baseline characteristics with an inverse probability of treatment weighting (IPTW), the TARE-ineligible group showed shorter TTP [adjusted hazard ratio (aHR)=2.16, 95% confidence interval (CI)=1.14–4.07, *P*=0.02] and OS (aHR=1.80, 95% CI=0.85–3.80, *P*=0.12), although the latter was not statistically significant. The TARE-ineligible group had a significantly lower objective response rate than the TARE-eligible group (9.7% *vs*. 56.9%, *P*<0.001).

**Conclusion:**

TARE-ineligible patients had larger tumors and higher MoRAL scores than TARE-eligible patients. Treatment with TACE, owing to high LSF, was associated with a shorter TTP even after balancing tumor size and MoRAL scores.

## Introduction

Hepatocellular carcinoma (HCC) is the most common primary liver cancer, accounting for approximately 90% of all liver cancers. Annually, 850,000 patients are newly diagnosed with HCC, which is the second leading cause of cancer-related deaths worldwide (1). Several curative and palliative treatments are available for HCC and are selected based on tumor stage, liver function and patient performance (2).

Transarterial radioembolization (TARE) is an internal radiation therapy which administrates yttrium-90 (^90^Y)-labelled microsphere emitting β-rays *via* a tumor feeding artery (3, 4). TARE provides better treatment outcomes with longer time to progression (TTP) (5) or overall survival (OS) than conventional transarterial chemoembolization (TACE) (6, 7), and is comparable to surgical resection in patients with a single large HCC (8).

Nevertheless, some patients do not undergo TARE treatment for a variety of reasons. High lung-shunt fraction (LSF) is the leading cause of TARE-ineligibility (9). Other minor causes of TARE-ineligibility include HCC adjacent to bowel adhesion as a result of prior intraabdominal surgery or procedure, considerable arterioportal shunt that may result in extrahepatic deposition of radioactive microspheres, and poor liver function (10, 11). High-energy β-rays may cause fatal radiation lung injury and severe radiation pneumonitis if substantial numbers of ^90^Y particles pass through shunts and reach the lung (12, 13). Thus, TARE is highly contraindicated in patients with high LSF, while a pretreatment simulation study before TARE treatment is required to evaluate TARE eligibility, unlike TACE. The lung shunt level is estimated using LSF of technetium-99m (^99m^Tc)-labeled macroaggregated albumin (MAA) scintigraphy (14). Patients who are ineligible for TARE using this pretreatment test are treated with other modalities, mostly TACE. Because the majority of TARE-eligible patients are Barcelona Clinic Liver Cancer (BCLC) B. In general, these BCLC B patients are unsuitable for hepatic resection and indication for TACE (15, 16). High LSF is not a contraindication for TACE since the risk of pulmonary complication due to intratumoral shunting is extremely rare (17). In addition, TACE is performed prior to TARE for patients with high initial LSF to lower LSF in real-world clinical practice (18). This indicates that TACE may be performed safely on patients with high LSF and could successfully embolize abnormally dilated vessels within HCC (19).

In this study, we aimed to compare the outcomes between patients who were eligible to TARE and underwent TARE and those who were ineligible to TARE and underwent TACE as an initial treatment for HCC.

## Materials and methods

### Patients

**Figure 1:**
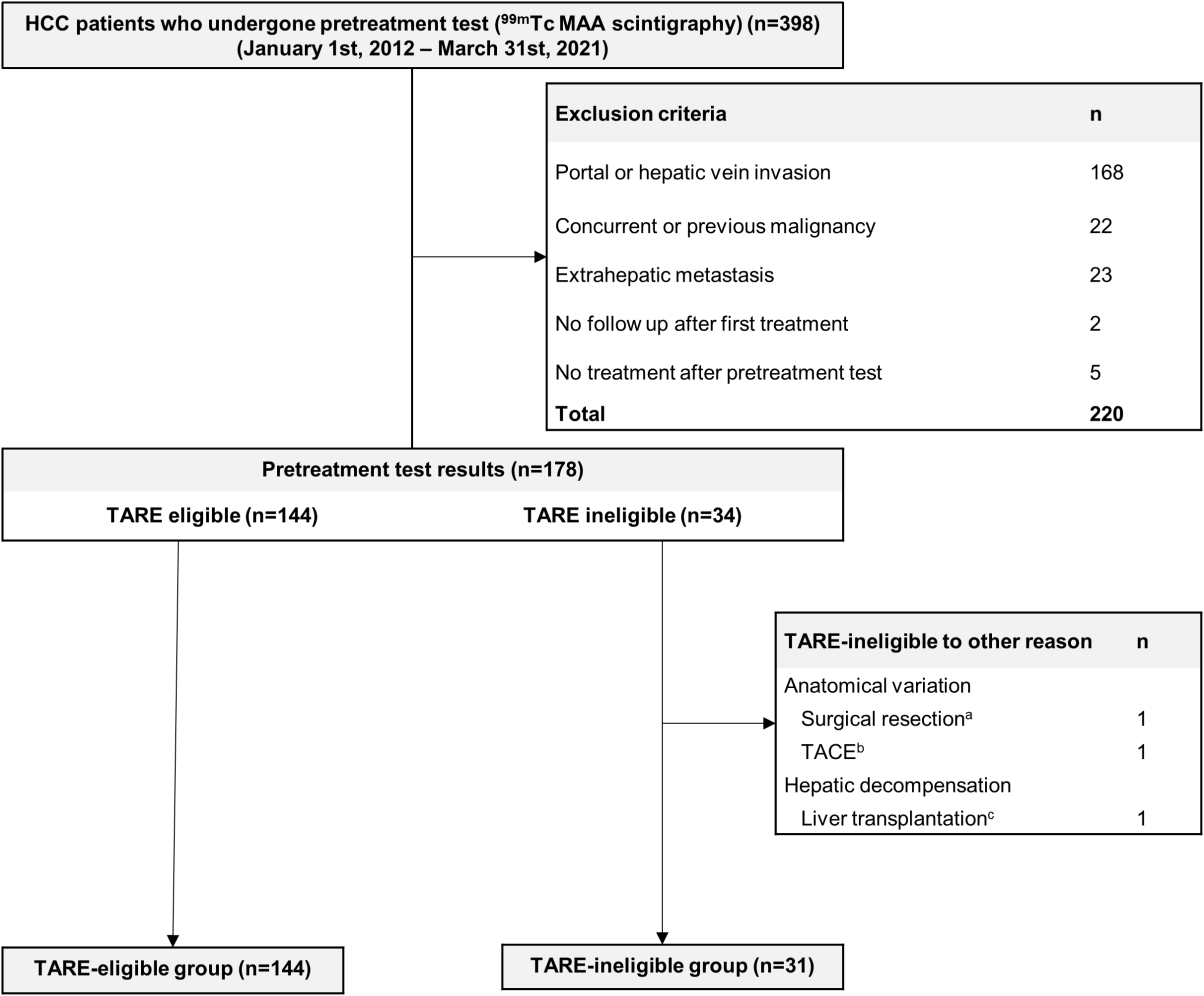
Flow Chart. A total of 398 HCC patients undergone pretreatment 99mTc MAA scintigraphy and 223 patients were excluded. A total of 175 patients were analyzed. ^a^The patient underwent a right posterior sectionectomy and caudate lobectomy due to the difficulty in targeting HCC (HCC feeding artery branched from pancreatic artery). ^b^TACE was performed on patient due to anatomical variance (left hepatic artery branching from left gastric artery causing difficult to target). ^c^Due to poor liver function, the patient received salvage living donor liver transplantation (Child-Pugh score 10). ^99m^Tc, technetium-99m; HCC, hepatocellular carcinoma; MAA, macroaggregated albumin; TACE, transarterial chemoembolization; TARE, transarterial radioembolization.

Consecutive patients with HCC who underwent pretreatment simulation studies as candidates for TARE, between September 2009 and March 2021 in a tertiary referral center (Seoul National University Hospital, Seoul, South Korea), were eligible for inclusion. Among eligible patients, those who had low LSF and underwent TARE or who had high LSF and underwent TACE were included. A HCC diagnosis was made according to radiographic or histological findings and followed American Association for the Study of Liver Disease guidelines (15). Exclusion criteria: (i) tumor thrombosis in the portal or hepatic vein, (ii) extrahepatic metastasis, (iii) poor liver function classified as Child-Pugh class C, (iv) poor performance status with a European Cooperative Oncology Group score ≥3, and (v) previous or current malignancies except HCC. Patients whose small HCC was treated with TARE without a pretreatment simulation test were also excluded. Patients who were ineligible for TARE due to anatomical variation (n=2, 5.9% of TARE-ineligible patients) and acute hepatic decompensation (n=1, 2.9% of TARE-ineligible patients) were also excluded (**Figure 1**). HCC-related factors (tumor size, BCLC stage) and laboratory data were collected retrospectively (platelet count, aspartate aminotransferase levels, alanine aminotransferase levels, prothrombin time, and albumin, etc.). Model to predict tumor recurrence after living donor transplantation (MoRAL) scores were calculated to estimate the prognosis of HCC: MoRAL score = 11·√protein induced by vitamin K absence-II (PIVKA-II) + 2·√alpha-fetoprotein (AFP). The MoRAL score according to reports, indicates the MoRAL score reflects the aggressiveness and burden of HCC tumors, and is associated with clinical outcomes following various treatments, including TACE and radiofrequency ablation (20–22).

The study was performed in accordance with the Declaration of Helsinki and approved by the Institutional Review Board of Seoul National University Hospital (No: H-2101-093-1189). Written informed consent from patients was waived because clinical data were anonymously analyzed.

### Procedures

For TARE candidates, pretreatment simulation tests were performed using hepatic angiography and ^99m^Tc-labeled MAA (Curium Pharma, London, United Kingdom) scintigraphy. Angiographic evaluations identified feeding arteries supplying the tumor and also non-target arteries that may cause aberrant ^90^Y deposition. By injecting ^99m^Tc-MAA into the optimal position in hepatic arteries, LSF was assessed. The recommended limit was either a lung dose >30 Gy/treatment or a cumulative lung dose of 50 Gy in TheraSphere^®^ (Boston Scientific, Natick, MA) or 20% of lung shunting for SIR-Spheres^®^ (Sirtex Medical, Lane Cove, Australia) (23, 24).

TARE was performed by two experienced interventional radiologists (H.C.K. and M.L.) who had >10 years of experience. Radioactive microspheres (TheraSphere^®^ and SIR-Spheres^®^) were infused according to previous protocol (23, 25). The mean target tissue dose by single compartment dosimetry ranged between 80–360 Gy for glass microspheres. When the patients had good liver function (Child-Pugh class A) and at least 30% of whole liver volume can be saved from irradiation, boosted radioembolization (>150 Gy of mean target tissue dose) was performed to enhance tumor response. Partition dosimetry was adopted for resin microspheres, normal liver dose was kept under 60Gy and absorbed tumor dose ranged between 120–360 Gy.

For TARE-ineligible patients, either conventional or drug-eluting bead (DEB)-TACE was performed according to the operator’s decision based on multiple factors (e.g., the insurance policy of South Korea, the actual cost of the procedure, etc.). Both conventional and DEB-TACE were conducted superselectively utilizing a microcatheter with 1.7–2.0 F tip (Progreat Lambda or Alpha; Terumo, Tokyo, Japan) under cone beam CT guidance. In conventional TACE, an emulsion comprising 2–10 mL of iodized oil (Lipiodol; Guerbet, Roissy, France) and 10–50 mg doxorubicin hydrochloride powder (Adriamycin RDF; Ildong Pharmaceutical Co., Seoul, Korea) was slowly injected until tumors were completely stained. As an additional embolization, gelatin sponge particles (150–350 μm or 350–560 μm) was infused until near-stasis was achieved. In DEB-TACE, one or two vials of DEB agent (DC Bead; Boston Scientific, Natick, MA) were used per patient: 70–150 or 100–300 μm-sized DEB agent was administered to each patient based on the interventionist’s discretion. Similar to the gelatin sponge used in conventional TACE, DEB agent was slowly infused through tumor-feeding arteries until near-stasis was achieved. Each vial of DEB agent used in the DEB-TACE, was loaded with 50 mg of doxorubicin for 1 hour. This doxorubicin-loaded DEB agent was suspended in a mixture containing 25 mL of normal saline and 25 mL of iodinated contrast agent. No patient utilized anti-reflux device. Further specific TACE procedure is explained elsewhere (26–28). When residual tumor or disease progression occurred after initial HCC treatment, patients received additional treatments after shared discussion.

### Outcomes

Primary outcomes were time-to-progression (TTP) and OS, and the secondary outcome was objective response rate within 6 months after initial treatment with either TARE or TACE. Patient survival data of the patients were obtained from the Ministry of the Interior and Safety of Korea. Responses were assessed using modified Response Evaluation Criteria in Solid Tumors criteria (29). Treatment responses were assessed at 1, 3, and 6 months after initial HCC treatment using multiphase dynamic computed tomography or magnetic resonance imaging. All images were evaluated by one radiologist with >10 years of experience.

### Statistical analysis

Chi-squared or Fisher’s exact tests were used to compare categorical variables and the Mann-Whitney U test for continuous variables. OS and TTP were compared using the Kaplan-Meier method and log-rank test. The hazard ratio (HR) and its 95% confidence interval (CI) were estimated using the Cox proportional hazards model. Inverse probability of treatment weighting (IPTW) was applied to balance baseline characteristics between TARE-eligible and TARE-ineligible groups (30). The Cox proportional hazards model was used to evaluate independent survival risk factors. OS comparisons between TARE-eligible and TARE-ineligible groups and univariable and multivariable Cox analyses were performed in an IPTW-weighted cohort. Correlation between TTP and OS was analyzed using Kendall test.

Analyses were performed using R 4.2.0 (R Foundation for Statistical Computing, Vienna, Austria). All statistical tests were two-sided, and *P* values <0.05 were considered statistically significant.

## Results

### Study population

In total, 175 patients were included: 144 patients in the TARE-eligible group received TARE [glass microsphere (TheraSphere^®^), n=136; ^90^Y resin microsphere (SIR-Spheres^®^), n=8], whereas 34 patients were ineligible for TARE. Among the 34 TARE-ineligible patients, 31 patients were ineligible to TARE due to high LSF (TARE-ineligible group), two patients underwent other procedures (hepatic resection and TACE) due to technical difficulty and one patient was not suitable for TARE due to abrupt deterioration of liver failure and underwent salvage living donor liver transplantation. All patients with high LSF underwent TACE: 28 patients underwent TACE and 3 underwent DEB-TACE. As shown (**Table 1**), significant differences in several baseline variables were observed, including tumor size, MoRAL scores, platelet counts, aspartate aminotransferase levels, alanine aminotransferase levels, prothrombin time, and albumin before IPTW. However, after applying IPTW, variables were generally well-balanced, including tumor size (**Table 1**).

**Table 1:** Baseline characteristics of the study population before and after IPTW.

Variables	Before IPTW			After IPTW		
	TARE-eligible (n = 144)	TARE-ineligible (n = 31)	*P*	TARE-eligible (n = 144)	TARE-ineligible (n = 31)	*P*
Age, years (IQR)	64.0 (57.0–72.0)	61.5 (52.8–69.2)	0.21	64.0 (57.0–72.0)	54 (50–70)	0.053
Sex, N (%)			0.11			0.39
Male	125 (84.0)	30 (96.8)		121 (84.0)	29 (90.7)	
Female	19 (16.0)	1 (3.2)		23 (16.0)	3 (9.3)	
Etiology, N (%)			0.99			0.79
HBV	89 (61.8)	19(61.3)		89 (61.8)	21 (67.7)	
HCV	7 (5.6)	2 (6.5)		7 (5.6)	1 (3.2)	
Alcohol	16 (11.1)	4 (12.9)		16 (11.1)	1 (3.6)	
HBV + alcohol	8 (4.9)	1 (3.2)		8 (4.9)	1 (3.6)	
HCV + alcohol	2 (1.4)	0 (0.0)		2 (1.4)	0 (0.0)	
HBV + HCV + alcohol	1 (0.7)	0 (0.0)		1 (0.7)	0 (0.0)	
Other	21 (14.6)	5 (16.1)		21 (14.6)	7 (22.6)	
Tumor size, cm (IQR)	7.8 (5.2–11.0)	13.8 (11.0–15.2)	<0.001	7.8 (5.2–11.0)	10.4 (7.3–12.3)	0.053
BCLC stage, N (%)			0.18			0.11
A	71 (49.3)	20 (64.5)		71 (49.3)	22 (71.0)	
B	73 (50.7)	11 (35.4)		73 (50.7)	9 (29.0)	
WBC,/μL (IQR)	6000 (4890–7720)	6000 (5140–7610)	0.97	6000 (4890–7720)	6000 (4480–8410)	0.88
Platelet, x 1,000/mm^3^ (IQR)	194.0 (156.0–252.0)	224.0 (154.0–346.0)	0.38	194.0 (156.0–252.0)	206.0 (101.0–239.0)	0.82
AST, IU/L (IQR)	43 (30–66)	98 (64–128)	<0.001	43 (30–66)	83 (42–99)	0.008
ALT, IU/L (IQR)	36 (24–54)	64 (41–90)	0.04	36 (24–54)	67 (43–79)	0.055
Total Bilirubin, mg/dL (IQR)	0.8 (0.6–1.1)	1.0 (0.9–1.4)	0.01	0.8 (0.6–1.1)	0.9 (0.8–1.2)	0.14
PT, INR (IQR)	1.0 (1.0–1.1)	1.1 (1.0–1.2)	0.002	1.0 (1.0–1.1)	1.1 (1.0–1.1)	0.052
Albumin, g/dL (IQR)	4.2 (3.9–4.4)	3.9 (3.5–4.2)	0.005	4.2 (3.9–4.4)	3.9 (3.4–4.4)	0.12
AFP, ng/mL (IQR)	14.2 (5.1–142.0)	1030.0 (11.3–10600)	0.22	14.2 (5.1–142.0)	137.4 (6.5–1263.0)	0.82
PIVKA, mAU/mL (IQR)	1260 (160–9300)	11500 (2240–54500)	0.01	1260 (160–9300)	2478 (153–11503)	0.62
MoRAL score	413.3 (163.4–1215.1)	1385.8 (582.5–3031.4)	0.002	413.3 (163.4–1215.1)	552.2 (159.5.0–1385.8)	0.98
Child-Pugh class, N (%)			0.14			0.09
A	142 (98.6)	29 (93.5)		142 (98.6)	29 (93.5)	
B	2 (1.4)	2 (6.5)		2 (1.4)	2 (6.5)	
LSF, % (IQR)	3.94 (2.52–6.86)	21.70 (16.70–28.60)	<0.001	3.79 (2.51–6.83)	20.20 (9.26–28.68)	<0.001

Categorical variables are presented as percentages and continuous variables as the median [interquartile range (IQR)].

AFP, alpha-fetoprotein; ALT, alanine transaminase; AST, aspartate aminotransferase; BCLC, Barcelona Clinic Liver Cancer; HBV, hepatitis B virus; HCV, hepatitis C virus; INR, international normalized ratio; IPTW, inverse probability of treatment weighting; LSF, lung shunt fraction; MoRAL, model to predict tumor recurrence after living donor transplantation; PIVKA, protein induced by vitamin K absence or antagonist-II; PT, prothrombin time; TARE, transarterial radioembolization; WBC, white blood cell.

### Comparing of TTP and OS

**Figure 2:**
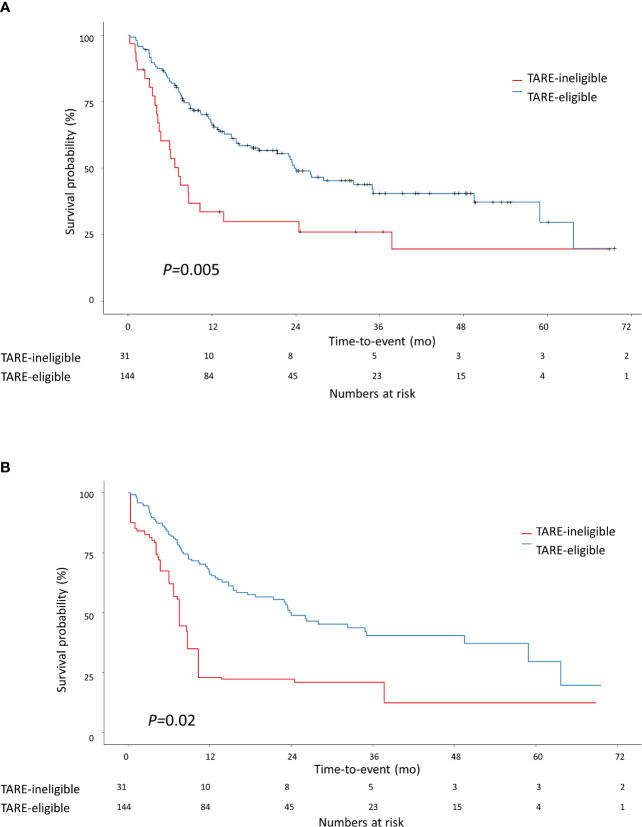
Kaplan-Meier methodology was used to estimate time-to-progression between TARE-eligible and TARE-ineligible groups **(A)** before applying IPTW and **(B)** after applying IPTW. Propensity score for IPTW were computed using the following variables: tumor size, MoRAL score, Child-Pugh class, and BCLC stage. BCLC, Barcelona Clinic Liver Cancer; IPTW, inverse probability of treatment weighting; MoRAL, model to predict tumor recurrence after living donor transplantation.

The median follow-up duration was 24.6 months [interquartile range (IQR)=13.5–37.4 months]. During follow-up, 97 patients experienced progression: 74 in the TARE-eligible group and 23 in the TARE-ineligible group. At month 6, patients in the TARE-eligible group had a lower probability of progression (17.5%) than those in the TARE-ineligible group (43.1%), and this difference persisted until month 24 (**Table 2A**). This latter had significantly shorter TTP (HR=1.96, 95% CI=1.22–3.14, log-rank *P=*0.005; **Figure 2**) than the former (**Table 3A**). This result was consistent with multivariable [adjusted hazard ratio (aHR)=1.94, 95% CI=1.10–3.43, *P*=0.02; **Table 3B**] and IPTW-weighted analyses (aHR=2.16, 95% CI=1.14–4.07, *P*=0.02).

Table 2The risk of (A) progression or (B) death at selected landmark time points.

**Table:** 

(A)				
	Month 6	Month 12	Month 18	Month 24
TARE-eligible	17.5%	33.9%	42.4%	51.0%
TARE-ineligible	43.1%	66.5%	29.8%	70.0%

**Table:** 

(B)				
	Month 6	Month 12	Month 18	Month 24
TARE-eligible	2.8%	8.5%	18.5%	21.0%
TARE-ineligible	16.1%	25.8%	35.9%	46.0%

Table 3Univariable and multivariable Cox analyses for the time-to-progression between TARE-eligible and TARE-ineligible groups before (A) and after (B) IPTW.

**Table:** 

(A)				
Variables	Univariable analysis		Multivariable analysis	
	HR (95% CI)	*P*	aHR (95% CI)	*P*
Sex				
Male	Ref			
Female	0.94 (0.52–1.73)	0.85		
Age, year	0.98 (0.97–1.00)	0.053	0.99 (0.97–1.00)	0.17
TARE-eligibility				
TARE-eligible	Ref		Ref	
TARE-ineligible	1.96 (1.22–3.32)	0.005	1.94 (1.10–3.43)	0.02
Tumor size, cm	1.06 (1.02–1.11)	0.005	0.97 (0.89–1.05)	0.46
MoRAL	1.0002 (1.0001–1.0004)	0.005	1.0001 (0.9999–1.0003)	0.49
AST, IU/L	1.006 (1.003–1.009)	<0.001	1.004 (1.000–1.009)	0.08
ALT, IU/L	1.000 (0.997–1.003)	0.95		
Child-Pugh class				
Class A	Ref		Ref	
Class B	2.45 (0.90–6.69)	0.07	1.64 (0.58–4.11)	0.35
BCLC stage				
BCLC A				
BCLC B	2.53 (1.67–3.84)	<0.001	2.54 (1.66–3.90)	<0.001
Platelet, x 1,000/mm^3^	1.001 (1.000–1.003)	0.10	1.002 (1.000–1.005)	0.04
LSF, %	1.02 (1.01–1.05)	0.002

**Table:** 

(B)				
Variables	Univariable analysis		Multivariable analysis	
	HR (95% CI)	*P*	aHR (95% CI)	*P*
Sex				
Male	Ref			
Female	0.93 (0.48–1.80)	0.84		
Age, year	0.97 (0.96–0.99)	0.01	0.98 (0.96–1.00)	0.09
TARE-eligibility				
TARE-eligible	Ref		Ref	
TARE-ineligible	2.30 (1.28–4.11)	0.01	2.16 (1.14–4.07)	0.02
Tumor size, cm	1.06 (1.01–1.11)	0.03	1.00 (0.91–1.09)	0.93
MoRAL	1.0001 (0.9999–1.0003)	0.35	0.9999 (0.9996–1.0003)	0.75
AST	1.007 (1.003–1.011)	0.001	1.006 (1.003–1.010)	0.001
ALT	1.000 (0.996–1.004)	1.00		
Child-Pugh class				
Class A	Ref		Ref	
Class B	1.98 (1.230–3.02)	0.002	1.30 (0.70–2.42)	0.40
BCLC stage				
BCLC A	Ref		Ref	
BCLC B	1.76 (1.03–2.99)	0.04	2.05 (1.28–3.27)	0.003
Platelet, x 1,000/mm^3^	1.001 (0.999–1.003)	0.35		
LSF, %	1.01 (0.99–1.04)	0.30		

AFP, alpha-fetoprotein; aHR, adjusted hazard ratio; ALT, alanine transferase; AST, aspartate transaminase; BCLC, Barcelona Clinic Liver Cancer; CI, confidence interval; HR, hazard ratio; IPTW, inverse probability of treatment weighting; LSF, lung shunt fraction; MoRAL, model to predict tumor recurrence after living donor transplantation; TARE, transarterial radioembolization.

**Figure 3:**
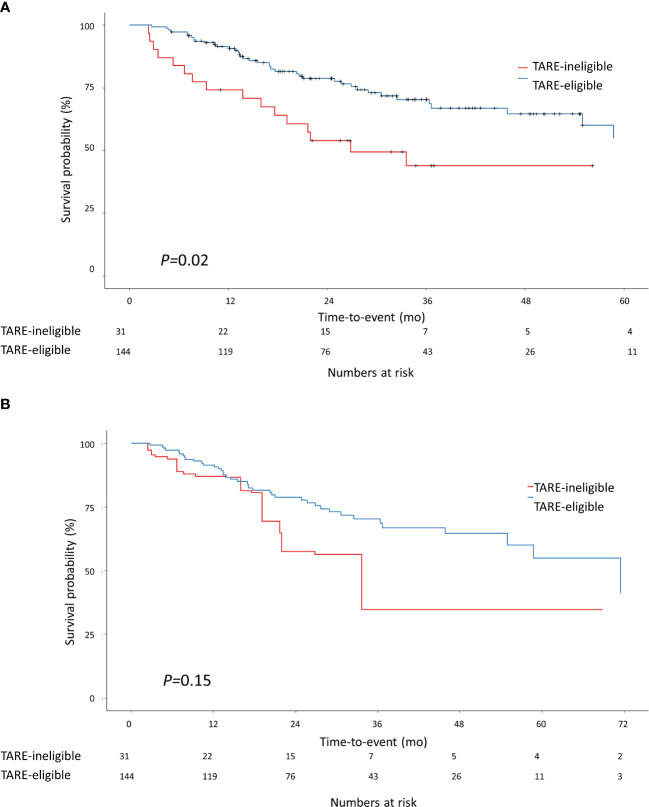
Kaplan-Meier methodology was used to estimate overall survival between the TARE-eligible and TARE-ineligible groups **(A)** before applying IPTW and **(B)** after applying IPTW. Propensity score for IPTW were computed using the following variables: tumor size, MoRAL score, Child-Pugh class, and BCLC stage. BCLC, Barcelona Clinic Liver Cancer; CI, confidence interval; IPTW, inverse probability of treatment weighting; MoRAL, model to predict tumor recurrence after living donor transplantation.

During the study period, 57 patients died: 41 in the TARE-eligible group and 16 in the TARE-ineligible group. At month 6, patients in the TARE-ineligible group had a higher risk of death (16.1%) than those in the TARE-eligible group (2.8%), and this risk persisted until month 24 (**Table 2B**). From univariable analysis of the crude population, the TARE-ineligible group had a shorter OS than the TARE-ineligible group (HR=1.87, 95% CI=1.04–3.36, *P*=0.03; **Figure 3A**). In multivariable analysis, the risk of death was similar in the TARE ineligible group and in the TARE-eligible group (aHR=1.28, 95% CI=0.66–2.51, P=0.46; **Table 4A**). After using IPTW, similar results maintained in both univariable (HR=1.79, 95% CI=0.92–3.47, log-rank *P*=0.15; **Figure 3B**) and multivariable (aHR=1.80, 95% CI=0.85–3.80, *P*=0.12; **Table 4B**) analyses. Between recurrence and death, we identified a significant correlation (by Kendall test, Z=11.00, tau=0.56, *P*<0.001).

Table 4Univariable and Multivariable Cox analyses for overall survival between TARE-eligible and TARE-ineligible groups before (A) and after (B) IPTW.

**Table:** 

(A)				
Variables	Univariable analysis		Multivariable analysis	
	HR (95% CI)	*P*	aHR (95% CI)	*P*
Sex				
Male	Ref			
Female	1.16 (0.55–2.46)	0.70		
Age, year	1.005 (0.983–1.027)	0.66		
TARE-eligibility				
TARE-eligible	Ref		Ref	
TARE-ineligible	1.87 (1.04–3.36)	0.04	1.28 (0.66–2.51)	0.46
Tumor size, cm	1.12 (1.05–1.18)	<0.001	1.06 (0.98–1.14)	0.17
MoRAL score	1.0004 (1.0002–1.0006)	<0.001	1.0002 (0.9999–1.0005)	0.24
AST, IU/L	1.005 (1.003–1.008)	<0.001	1.003 (0.999–1.005)	0.20
ALT, IU/L	1.001 (0.999–1.004)	0.33		
Child-Pugh class				
Class A	Ref		Ref	
Class B	3.84 (1.38–10.66)	0.01	2.87 (1.01–8.12)	0.047
BCLC stage				
BCLC A	Ref		Ref	
BCLC B	2.01 (1.18–3.43)	0.01	1.92 (1.09–3.37)	0.02
Platelet, x 1,000/mm^3^	1.002 (0.999–1.004)	0.07		
LSF, %	1.022 (0.997–1.047)	0.08

**Table:** 

(B)				
Variables	Univariable analysis		Multivariable analysi	
	HR (95% CI)	*P*	aHR (95% CI)	*P*
Sex				
Male	Ref			
Female	1.37 (0.63–2.98)	0.42		
Age, years	0.986 (0.960–1.013)	0.32		
TARE-eligibility				
TARE-eligible	Ref		Ref	
TARE-ineligible	1.79 (0.92–3.47)	0.09	1.80 (0.85–3.80)	0.12
Tumor size, cm	1.09 (1.03–1.16)	0.004	1.03 (0.94–1.14)	0.47
MoRAL score	1.0003 (1.0000–1.0006)	0.06	1.0001 (0.9998–1.0005)	0.48
AST, IU/L	1.005 (1.002–1.008)	<0.001	1.004 (1.000–1.007)	0.04
ALT, IU/L	1.001 (0.998–1.004)	0.58		
Child-Pugh class				
Class A	Ref		Ref	
Class B	3.09 (1.90–5.03)	<0.001	2.91 (1.45–5.85)	0.002
BCLC stage				
BCLC A	Ref		Ref.	
BCLC B	1.80 (0.91–3.57)	0.09	2.02 (0.98–4.16)	0.06
Platelet, x 1,000/mm^3^	1.002 (0.998–1.005)	0.18		
LSF, %	1.00 (0.96–1.03)	0.88		

AFP, alpha-fetoprotein; aHR, adjusted hazard ratio; ALT, alanine transferase; AST, aspartate transaminase; BCLC, Barcelona Clinic Liver Cancer; CI, confidence interval; HR, hazard ratio; IPTW, inverse probability of treatment weighting; LSF, lung shunt fraction; MoRAL, model to predict tumor recurrence after living donor transplantation; TARE, transarterial radioembolization.

We performed subgroup analysis based on different factors (i.e., age, BCLC stage, Child-Pugh class, tumor size, and MoRAL score) and similar results regarding TTP and OS were reproduced (**Table 4**).

### Tumor response to initial treatment

During follow-up for 6 months after the initial treatment, 58 (40.3%), 24 (16.7%), 59 (41.0%), and 3 (2.1%) patients achieved best overall responses for CR, PR, SD, and PD, respectively in the TARE-eligible group. In the TARE-ineligible group, 3 (9.7%), 0 (0.0%), 25 (80.6%), and 3 (9.7%) patients achieved the best responses for CR, PR, SD, and PD respectively. The TARE-ineligible group had a significantly lower objective response rate than the TARE-eligible group (9.7% *vs*. 56.9%, *P*<0.001), while the disease control rate did not show significant differences between groups (90.3% *vs*. 97.9%, *P*=0.07).

Patients with residual tumors or disease progression after initial HCC treatment underwent different treatments (**Table 5**). The TARE-eligible group had similar number (*P*=0.19) of subsequent treatments (2.0 times, IQR=1.0–4.0 times) as the TARE-ineligible group (3.0 times, IQR=1.0–5.5 times).

Table 5Subgroup analysis of (A) time-to-progression and (B) overall survival.

**Table:** 

(A)					
	Events/Patients		*vs*. TARE-ineligible		
	TARE-eligible	TARE-ineligible	HR (95% CI)	*P*	*P*_interaction_
Age					0.23
≤65 years	39/77	17/21	2.42 (1.36–4.32)	0.003	
>65 years	35/67	6/10	1.30 (0.54–3.10)	0.56	
Tumor size^a^					0.37
≤8.3 cm	39/83	5/6	2.60 (1.02–6.67)	0.046	
>8.3 cm	35/61	18/25	1.54 (0.87–2.73)	0.14	
MoRAL score^b^					0.59
≤200.6	16/39	3/5	1.42 (0.41–4.90)	0.58	
>200.6	58/105	20/26	2.03 (1.22–3.39)	0.007	
Child-Pugh class					0.66
A	72/142	21/39	1.86 (1.14–3.05)	0.01	
B	2/2	2/2	2.95 (0.41–21.10)	0.28	
BCLC stage					0.69
A	25/71	13/20	2.57 (1.30–5.06)	0.006	
B	49/73	10/11	2.11 (1.07–4.20)	0.03

**Table:** 

(B)					
	Events/Patients		*vs*. TARE-ineligible		
	TARE eligible	TARE-ineligible	HR (95% CI)	*P*	*P*_interaction_
Age					0.43
≤65	19/77	11/21	2.34 (1.10–4.97)	0.03	
> 65	22/67	5/10	1.43 (0.54–3.80)	0.47	
Tumor size^a^				0.28	
≤8.3 cm	17/83	4/6	2.60 (1.02–6.67)	0.046	
>8.3 cm	24/61	12/25	1.54 (0.87–2.73)	0.14	
MoRAL score^b^					0.32
≤200.6	9/39	1/5	0.76 (0.10–6.03)	0.79	
>200.6	32/105	15/26	2.02 (1.09–3.74)	0.03	
Child-Pugh class				0.78	
A	39/142	14/29	1.74 (0.94–3.23)	0.08	
B	2/2	2/2	2.33 (0.33–17.00)	0.39	
BCLC stage					0.97
A	14/71	9/20	2.22 (0.95–5.19)	0.07	
B	27/73	7/11	2.27 (0.98–5.23)	0.054	

a Tumor size = 8.3 cm was the median value for the study population.

b MoRAL score = 200.6 was the lower 75th percentile MoRAL score value for the study population.

BCLC, Barcelona Clinic Liver Cancer; HR, hazard ratio; MoRAL, model to predict tumor recurrence after living donor transplantation; TARE, transarterial radioembolization.

## Discussion

In this study, we compared clinical outcomes between patients eligible and ineligible for TARE as an initial treatment for large HCC. The TARE-ineligible group tended to have larger tumor sizes and higher MoRAL scores than the TARE-eligible group. The TARE-ineligible group had shorter TTP than the TARE-eligible group in crude analysis, even after balancing baseline variables including tumor size and MoRAL score with IPTW. The risk of death was 35%–92% higher in the TARE-ineligible group than the TARE-eligible group, although it failed to reach statistical significance in multivariable and/or IPTW analyses. The objective response rate was significantly higher in the TARE-eligible group than the TARE-ineligible group. Our study was conducted in a single tertiary center where all treatments and decisions were made by highly experienced physicians and interventionists, something that would not have been possible in small-volume institutions. In terms of study design, these highly qualified specialists enable us to compare TACE and TARE treatments by minimizing the impact of human variables (low proficiency of TARE or TACE techniques).

As stated, patients in the TARE-ineligible group had larger tumors and higher MoRAL scores than the TARE-eligible group. As prognostic factors, large tumor size and high MoRAL scores in the TARE-ineligible group may account for shorter TTP or OS than the TARE-eligible group. Large HCC is associated with a poor prognosis (31, 32). Two components of MoRAL scores, AFP and PIVKA, are associated with aggressive tumor behaviors and poor clinical outcomes (33–35).

LSF may have functioned as a confounder since a higher LSF was associated with more aggressive tumor behavior as well as worse clinical outcomes (36) in previous studies and TARE-eligibility was directly related to LSF. In our study, however, after baseline characteristics were balanced using IPTW, there was no association between LSF and clinical outcomes (i.e., time to progression and overall survival). Based on the traditional definition of confounder (37), which is defined as a pre-exposure variable associated with both exposure and outcome, LSF was not a confounder in our study. However, due to the inadequacy of our study design to investigate the confounding effect of LSF, validation requires additional study.

In previous studies, the OS of patients treated with TARE was longer than in patients treated with conventional TACE (6, 7). Moreover, patients treated with conventional TACE, because they were ineligible for TARE had worse prognoses than those eligible for TARE and treated with TARE. Thus, early systemic therapy must be considered for these patients. This strategy is similar to the treatment strategy for TACE-refractory patients (38). TARE-ineligible patients share similar features to TACE-refractory patients, such as high MoRAL scores or high AFP levels and large tumor sizes (39). Similar TACE-refractoriness in TARE-ineligible patients is to be expected and early transition to systemic therapy could improve patient outcomes.

Our study had several limitations. First, due to its retrospective nature, unintended biases may have been introduced. We applied IPTW and multivariable analysis to offset differences by balancing the characteristics of both groups to minimize the selection bias, and multiple subgroup analyses to validate our points. Also, since only the outcome of TACE for TARE-ineligible patients with high LSF was evaluated, it is critical not to overinterpret this as a sign that TACE is inferior.

In conclusion, TARE-ineligible HCC patients treated with TACE had shorter TTP than TARE eligible patients. Further study might be required to determine whether other treatment strategies, such as surgical resection and systemic therapy could improve outcome in TARE-ineligible patients with a high LSF.

## Data availability statement

The data that support the findings of this study are available from the corresponding authors, upon reasonable request.

## Ethics statement

The studies involving human participants were reviewed and approved by the Institutional Review Board of Seoul National University Hospital. The ethics committee waived the requirement of written informed consent for participation.

## Author contributions

The corresponding authors (J-HL and H-CK) had full access to all the data in the study and takes responsibility for the integrity of the data and the accuracy of the data analysis. Conceptualization and methodology: HC, SC, J-HL, and H-CK; software: SC and HC; validation: J-HL and H-CK; formal analysis: SC and HC; data curation and investigation: SC, HC, HS, JP, JK, JH, and MH; resources: MP, YL, SY, ML, YK, JP, J-HY, JC, J-HL, and H-CK; writing – original draft preparation: SC; writing – review and editing: HC and J-HL; visualization: SC and MP; supervision: J-HL and H-CK; project administration: J-HL and H-CK. All authors contributed to the article and approved the submitted version.
